# Genomic Characterization of Two Shiga Toxin–Converting Bacteriophages Induced From Environmental Shiga Toxin–Producing *Escherichia coli*

**DOI:** 10.3389/fmicb.2021.587696

**Published:** 2021-02-25

**Authors:** Yujie Zhang, Yen-Te Liao, Alexandra Salvador, Vivian C. H. Wu

**Affiliations:** Produce Safety and Microbiology Research Unit, US Department of Agriculture, Agricultural Research Service, Western Regional Research Center, Albany, CA, United States

**Keywords:** Stx-converting bacteriophages, Shiga toxin-producing *Escherichia coli*, comparative genomics, genetic diversity, virulence gene transfer

## Abstract

Shiga toxin (Stx), encoded by *stx* genes located in prophage sequences, is the major agent responsible for the pathogenicity of Shiga toxin-producing *Escherichia coli* (STEC) and is closely associated with the development of hemolytic uremic syndrome (HUS). Although numerous Stx prophage sequences have been reported as part of STEC bacterial genomes, the information about the genomic characterization of Stx-converting bacteriophages induced from STEC strains is relatively scarce. The objectives of this study were to genomically characterize two Stx-converting phages induced from environmental STEC strains and to evaluate their correlations with published Stx-converting phages and STEC strains of different origins. The Stx1-converting phage Lys8385Vzw and the Stx2-converting phage Lys19259Vzw were induced from *E. coli* O103:H11 (RM8385) and *E. coli* O157:H7 (RM19259), respectively. Whole-genome sequencing of these phages was conducted on a MiSeq sequencer for genomic characterization. Phylogenetic analysis and comparative genomics were performed to determine the correlations between these two Stx-converting phages, 13 reference Stx-converting phages, and 10 reference STEC genomes carrying closely related Stx prophages. Both Stx-converting phages Lys8385Vzw and Lys19259Vzw had double-stranded DNA, with genome sizes of 50,953 and 61,072 bp, respectively. Approximately 40% of the annotated coding DNA sequences with the predicted functions were likely associated with the fitness for both phages and their bacterial hosts. The whole-genome–based phylogenetic analysis of these two Stx-converting phages and 13 reference Stx-converting phages revealed that the 15 Stx-converting phages were divided into three distinct clusters, and those from *E. coli* O157:H7, in particular, were distributed in each cluster, demonstrating the high genomic diversity of these Stx-converting phages. The genomes of Stx-converting phage Lys8385Vzw and Lys19259Vzw shared a high-nucleotide similarity with the prophage sequences of the selected STEC isolates from the clinical and environmental origin. The findings demonstrate the genomic diversity of Stx-converting phages induced from different STEC strains and provide valuable insights into the dissemination of *stx* genes among *E. coli* population via the lysogenization of Stx-converting phages.

## Introduction

Shiga toxin–producing *Escherichia coli* (STEC) are notorious foodborne pathogens that cause several human diseases, such as diarrhea, hemorrhagic colitis, and hemolytic uremic syndrome (HUS) (Heiman et al., 2015; White et al., 2016). The gastrointestinal tract of ruminant animals has been reported to be the primary reservoir of STEC, likely causing the contamination of products of animal origin and the surrounding environment, such as cross-contamination on produce farms (Sharma et al., 2019). Aside from *E. coli* O157:H7, certain non-O157 STEC strains are also frequently associated with foodborne outbreaks in recent years (Frank et al., 2011; Jenkins et al., 2015; Valilis et al., 2018). For example, several outbreaks caused by *E. coli* O157:H7 have been reported in the United States between 2013 and 2020 and have been related to the contamination of leafy greens, beef products, and butter (CDC, 2014, 2017, 2018; USDA, 2020). Additionally, four foodborne outbreaks were caused by some of the most frequently occurring non-O157 STEC serogroups in the United States—including O26, O121, and O103—from 2019 to 2020, and STEC O103, in particular, was attributed to three foodborne outbreaks associated with contaminated Northfork bison, ground beef, and clover sprouts, causing 50 hospitalization and two patients with HUS (CDC, 2019, 2020).

Shiga toxin (Stx), encoded by *stx* genes, is one of the primary virulence factors of STEC strains and can exhibit cytotoxicity to various target cells, such as endothelial cells (Melton-Celsa, 2014). *Stx1* and *stx2* genes are categorized into three subtypes (*stx1a*, s*tx1c*, *stx1d*) and seven subtypes (*stx2a* through *stx2g*), respectively, based on nucleotide sequences (Krüger and Lucchesi, 2015). Recently, more *stx* gene subtypes were identified along with the development of whole-genome sequencing technology and the subsequent sequencing of various STEC strains. For instance, Bai et al. found a novel *stx2* subtype, classified as *stx2h*, from a pathogenic *E. coli* strain isolated from wild Himalayan marmot (Bai et al., 2018). Previous studies have found that STEC strains harboring the *stx2a* subtype were the strains that are most frequently associated with the development of HUS in patients relative to the other subtypes (Friedrich et al., 2002; Persson et al., 2007; Krüger et al., 2018). Moreover, *stx* genes were confirmed to be located on Stx prophage sequences within the bacterial chromosome (Plunkett et al., 1999; Tyler et al., 2004; Tóth et al., 2016). Thus, the characterization of Stx prophage facilitates understanding of the pathogenicity of STEC strains. Cooper et al. found two outbreak *E. coli* O145:H28 strains originally shared a close evolutionary relationship with *E. coli* O157:H7 strains, such as *E. coli* Sakai and EDL933, but were subsequently evolved to lineage-independent pathogenicity by acquiring additional virulence factors, including a Stx2a prophage and a large virulence plasmid (Cooper et al., 2014). Forde et al. evaluated two STEC O111:H- strains isolated from HUS patients related to a foodborne outbreak in Australia and found that one STEC O111 strain containing a total of four Stx prophages was more virulent than the other strain that harbored two Stx prophages (Forde et al., 2019).

Stx prophages can be induced from STEC strains by different external stresses and subsequently released in the environment as Stx-converting phages, which are capable of infecting other susceptible strains and pose a risk of virulence gene transfer. Bacteriophage 933W, a well-studied Stx-converting phage, can self-induce from *E. coli* O157:H7 EDL933 strain and subsequently infect other susceptible *E. coli* strains to form a new lysogen (Bullwinkle et al., 2012). Stx-converting phages are also highly associated with the evolution of pathogens and result in the increased pathogenicity of these pathogens. During the 2011 German outbreak of contaminated sprouts, a new pathogenic *E. coli* O104:H4 strain was isolated and identified to harbor major virulence factors from both enteroaggregative *E. coli* (EAEC) and enterohemorrhagic *E. coli* (EHEC), causing 54 deaths (Frank et al., 2011). The enteroaggregative hemorrhagic *E. coli* (EAHEC) O104:H4 outbreak strain was demonstrated to evolve from EAEC through the acquisition of a Stx2a-converting phage (Beutin and Martin, 2012). Furthermore, Bai et al. found that several clinical isolates from diarrheal patients were STEC/enterotoxigenic *E. coli* hybrids, which were also related to the acquisition of Stx-converting phages (Bai et al., 2019). These findings suggest that the emergence and the evolution of STEC strains are closely associated with the transduction of Stx-converting phages.

Although numerous Stx prophages have been studied and shown to be one of the key elements of various complete STEC genome sequences, little is known about the genomic characterization of the Stx-converting phages induced from STEC strains (Plunkett et al., 1999; Smith et al., 2012; Grande et al., 2014; Duan et al., 2018). Here, we aimed to genomically characterize induced Stx1-converting phages Lys8385Vzw and Stx2-converting phage Lys19259Vzw and to evaluate their genetic correlation with known Stx-converting phages and STEC pathogens from different sources via comparative genomics.

## Materials and Methods

### Bacterial Strains and Bacteriophages

Two STEC strains—*E. coli* O103:H11 strain RM8385 and *E. coli* O157:H7 strain RM19259—were initially isolated from cattle feces and river samples, respectively, and stored at the culture collections of the Produce Safety and Microbiology Research Unit in the US Department of Agriculture–Agricultural Research Service, Western Regional Research Center. Stx1-converting phage Lys8385Vzw and Stx2-converting phage Lys19259Vzw were induced from RM8385 and RM19259 strains, respectively, by use of mitomycin C (0.5 μg/mL), followed by single-plaque purification and phage morphology observation via transmission electron microscopy as previously described (Supplementary Figure S1; Zhang et al., 2020). An *E. coli* strain ATCC700078 (WG5) was used to propagate Stx-converting phages. All bacterial strains used in this study were stored at −80°C and grown in 10 mL Luria-Bertani (LB, Difco, Becton Dickinson, Sparks, MD) at 37°C with shaking at 175 rpm prior to use.

### Phage Propagation and DNA Extraction

Both Stx-converting phages—Lys8385Vzw and Lys19259Vzw—were enriched in 400 mL no-salt, soft top (NS-ST) broth (10 g bacto-tryptone, 5 g yeast extract, 10 mM MgSO_4_/L) with the host strain *E. coli* WG5 at 37°C with shaking at 100 rpm overnight for phage propagation. The propagated phages were filtered through a 0.22 μm membrane syringe filter, followed by concentration using 50K Amicon Ultra centrifugal filter columns (Merck Millipore, Schwalbach, Germany). After CsCl gradient ultracentrifugation at 131,300 × g for 24 h, the CsCl-treated phages were washed and resuspended in SM buffer using 50K Amicon Ultra centrifugal filter columns (Merck Millipore, Schwalbach, Germany). DNase I (100 U/mL) was added to the phage lysates, and samples were incubated at 37°C for 60 min to remove residual bacteria DNA. Phage DNA was extracted by Norgen phage DNA extraction kit (Norgen Biotek, Ontario, Canada) and purified using DNeasy PowerClean Pro Cleanup Kit (QIAGEN Sciences, Germantown, United States) per the manufacturer’s instruction.

### Phage Whole-Genome Sequencing

Phage DNA libraries were constructed as previously described (Zhang et al., 2019). Briefly, Nanodrop 8000 and Qubit 2.0 were used to determine the quality and concentration of phage DNA samples, respectively. Two hundred nanograms of DNA was fragmented to 550 bp with an M220 Focused Ultrasonicator (Covaris, Woburn, MA, United States) and used for DNA library construction with a TruSeq^®^ Nano DNA Library Prep Kit (Illumina, San Diego, CA, United States) per the manufacturer’s instructions. An Illumina MiSeq sequencing platform with MiSeq reagent kit v2 (500-cycle) was used for loading the libraries and sequencing, generating approximately 5 million and 3.8 million paired-end (2 × 250 bp) sequence reads for phage Lys8385Vzw and Lys19259Vzw, respectively.

### Phage Genome Assembly and Annotation

Assembly and annotation of the phage sequences were performed as previously described (Zhang et al., 2019). Briefly, quality control of the raw sequence reads was performed using FASTQC and Trimmomatic (Bolger et al., 2014), with a trimming quality threshold of Q30. *De novo* assembly of the remaining quality reads was performed using SPAdes version 3.13.0 on the Kbase Server with a contig length set to >10,000 bp (Nurk et al., 2013; Arkin et al., 2018), and three resulting contigs for Lys8385Vzw and two resulting contigs for Lys19259Vzw were obtained. The resulting contigs of each phage were subjected to blastn against the National Center for Biotechnology Information (NCBI) database^1^. Subsequently, the final two contigs from each phage, sharing high nucleotide sequence similarity with the reference Stx-converting phages/Stx prophages obtained from the NCBI database, were identified as Stx-converting phages Lys8385Vzw and Lys19259Vzw, respectively. The genome annotation was conducted using both Prokka (Seemann, 2014) and the RAST Server (Aziz et al., 2008). The annotation was confirmed and curated using Uniprot (Bateman et al., 2015), open reading frame finder (Geneious version 11.0.4), and BLASTP^2^. tRNA was predicted using tRNAscan-SE web server (Lowe and Chan, 2016). ResFinder (version 3.0) and VirulenceFinder (version 2.0) were used to screen for antibiotic resistance genes and virulence genes (Zankari et al., 2012; Joensen et al., 2014). The phage packaging mechanisms of Stx-converting phages Lys8385Vzw and Lys19259Vzw were analyzed via PhageTerm with the default setting (Garneau et al., 2017).

### Phylogenetic Analysis of 15 Stx-Converting Phages From Different Origins

Thirteen complete genome sequences of Stx-converting phages, including two *Shigella* phages, induced from the bacterial strains rather than the Stx prophage sequences extracted from bacterial genome sequences, were downloaded from the NCBI database and used to compare with the Stx-converting phages isolated in this study by phylogenetic analysis. Multiple sequence alignments were performed on the whole-genome sequences of Stx-converting phages with the MAFFT algorithm using Geneious (version 11.1.5). The phylogenetic tree was performed using MEGA-X with the maximum likelihood method and further visualized using the ITOL web server (Kumar et al., 2018; Letunic and Bork, 2019). GenBank accession numbers of all reference phages are listed in Supplementary Table S1.

### Comparative Analysis of Stx-Converting Phages, Stx Prophages, and STEC Strains

Based on the phylogenetic analysis, the four most closely related phages, including Lys8385Vzw, Stx-converting phage 1717, Lys19259Vzw, and bacteriophage 933W, were selected for further comparison of genomic characteristics. Comparative genomic analysis was performed using Artemis 18.0.3 and Easyfig_2.2.3, with a minimum nucleotide sequence identity of 70% (Carver et al., 2005; Sullivan et al., 2011).

Additionally, sequences between Stx-converting phage and the Stx prophage from their corresponding bacterial genomes were compared to evaluate genomic alterations during prophage induction from their bacterial hosts. Stx prophage sequences were predicted from the complete bacterial genomes of *E. coli* O103:H11 (RM8385) and *E. coli* O157:H7 (RM19259) using PHASTER, Prophage Hunter and Vibrant, and manually curated to avoid misprediction (Arndt et al., 2016; Song et al., 2019; Kieft et al., 2020). The resulting Stx prophage sequences were further used to compare with the Stx-converting phages Lys8385Vzw and Lys19259Vzw induced from the same host strains. The comparative genomics was visualized using Easyfig_2.2.3 with a minimum nucleotide sequence identity set to 70% (Sullivan et al., 2011; Joensen et al., 2014; Arndt et al., 2017).

Both complete genome sequences of phage Lys8385Vzw and Lys19259Vzw were subjected to blastn searches against the NCBI database^3^. As a result, 10 different STEC reference genomes were obtained based on the high nucleotide sequence identity of their Stx prophage sequences to either genome of phage Lys8385Vzw (*n* = 5) or Lys19259Vzw (*n* = 5). Subsequently, the sequences of each Stx-converting phage (Lys8385Vzw and Lys19259Vzw) and its five corresponding STEC reference genomes, which contained similar Stx prophage sequences, were compared and visualized with specific sequence segments of high similarity using BRIG with default settings (Alikhan et al., 2011). The accession numbers of STEC strains used in this study are listed in Supplementary Table S2.

### Nucleotide Sequence Accession Numbers

The complete genome sequences of Stx1-converting phage Lys8385Vzw and Stx2-converting phage Lys19259Vzw have been deposited in GenBank under the accession numbers of MT225100 and MT225101, respectively.

## Results

### General Genomic Features of Stx1-Converting Phage Lys8385Vzw

**FIGURE 1:**
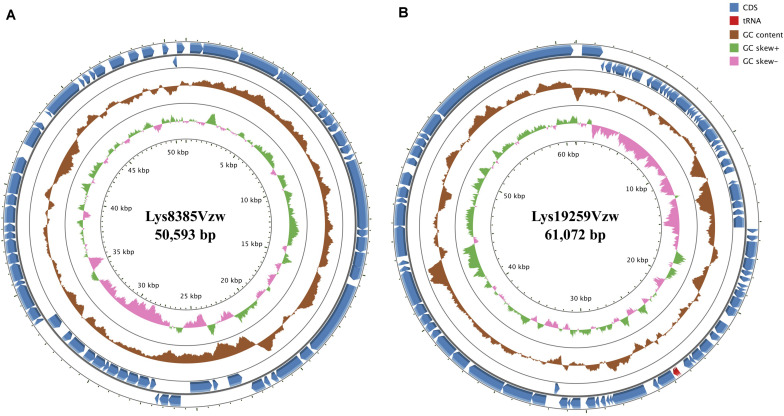
Circular genome maps of Stx1-converting phage Lys8385Vzw **(A)** and Stx2-converting phage Lys19259Vzw **(B)** using GCview server. The rings from inside out represent GC skew (green and pink), GC content (brown), and CDSs (blue). tRNAs (red) are only detected in Stx2-converting phage Lys19259Vzw **(B)**. The functions of the annotated CDSs are listed in Supplementary Tables S3, S4.

Phage Lys8385Vzw had double-stranded DNA with a genome size of 50,953 bp and an average G + C content of 51.6% (Figure 1A). A total of 68 coding DNA sequences (CDSs) were annotated, including 24 with unknown functions (hypothetical proteins) and 44 with predicted functions (Supplementary Table S3). A total of 17 CDSs encoded putative proteins that were associated with phage morphogenesis, including capsid protein, structural proteins, tail assembly protein, portal proteins, and tail proteins. Two CDSs with the predicted lysis protein in the phage Lys8385Vzw genome shared a high nucleotide sequence similarity with the CDSs annotated with spainin and holin in *Escherichia* phage BP-4795 (88.2% identity) and phage ArgO145 (100% identity), respectively, and the both CDSs were related to the lysis of bacterial cells and the release of phage progenies (Young, 2014). Additionally, two putative terminases were identified to be associated with the phage DNA packaging of bacteriophage (Catalano, 2000). Stx1-converting phage Lys8385Vzw also contained a CDS annotated with the predicted function of integrase, which was previously determined to assist with the integration of the phage genome into the bacterial chromosome (Groth and Calos, 2004). Additionally, six CDSs in phage Lys8385Vzw were predicted as the genetic regulatory functions, such as repressor, regulator, and antitermination protein Q, in particular, which were associated with the regulation of phage late-gene expression (Plunkett et al., 1999). Most importantly, phage Lys8385Vzw also contained the annotated CDS with regard to the type III secretion system effector NleG7 in addition to the virulence factor of *stx1a*. No antibiotic-resistant gene was found in the genome of phage Lys8385Vzw.

### General Genomic Features of Stx2-Converting Phage Lys19259Vzw

Phage Lys19259Vzw contained double-stranded DNA 61,072 bp in length, with an average G + C content of 49.2% (Figure 1B). Eighty-five CDSs were annotated in the genome of Lys19259Vzw, and 38 of which were with the predicted functions (Supplementary Table S4). The CDSs related to phage morphogenesis, such as phage capsid proteins and tail fiber proteins, were also predicted in the phage genome. Additionally, phage Lys19259Vzw carried the CDSs with the putative functions associated with cell lysis, including holin and lysis protein (Young, 2014). Furthermore, the lambda red recombineering system-coding sequences, including phage exonuclease Exo, phage recombination protein Bet, and regulator host-nuclease inhibitor protein Gam that were associated with phage DNA recombination, were annotated in the phage genome (Caldwell and Bell, 2019). Lys19259Vzw also contained the CDS annotated with the predicted function of integrase. A total of 10 CDSs with the predicted regulatory functions were found in phage Lys19259Vzw genome, including antitermination protein Q, Cro, CI, CII, CIII, and N, which were related to lysogenic conversion (Casjens and Hendrix, 2015). Phage Lys19259Vzw carried *stx2a* as the virulent factor and three tRNAs, including two tRNAs-*Arg* and one tRNA-*Met*, around the *stx2a* gene. No antibiotic-resistant genes were found in the phage genome.

### Phylogenetic Tree Reveals the Diversity of Stx-Converting Phages

**FIGURE 2:**
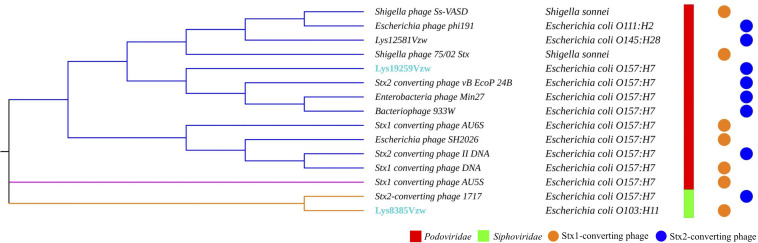
Maximum likelihood phylogenetic analysis of two Stx-converting phages, Lys8385Vzw and Lys19259Vzw, and 13 reference Stx-converting phages from the NCBI database. Different line colors represent different clusters. The phages highlighted with blue font were sequenced in this study. The bacterial strains next to each phage are the hosts from which the Stx-converting phages were induced. Phage morphologies for the *Siphoviridae* and *Podoviridae* families are indicated with red and green color bars, respectively. The circle with orange or blue color indicates the *stx* gene encoded in the Stx-converting phages.

Fifteen Stx-converting phage sequences, consisting of 13 reference phage genomes obtained from NCBI, phage Lys8385Vzw, and phage Lys19259Vzw, were used to determine the evolutionary relationships. The results showed that these 15 Stx-converting phages were categorized into three distinct clusters: cluster 1, cluster 2, and cluster 3 (Figure 2). Cluster 1 was the main cluster and was composed of 12 Stx-converting phages, including Lys19259Vzw, 933W, and two *Shigella* phages. Cluster 2 contained only Stx1-converting phage AU5s, which was isolated from a clinical *E. coli* O157 strain in Australia, and cluster 3 included Lys8385Vzw and Stx2-converting phage 1717. The phylogenetic result coincided with the classification of phage morphology: all of the phages classified in cluster 1 and cluster 2 belonged to the *Podoviridae* family, and the phages classified in cluster 3 belonged to the *Siphoviridae* family. Moreover, cluster 1 was further divided into three different clades: clade 1, clade 2, and clade 3. Clade 1 contained two *Shigella* phages, Ss-VASD and 75/02 Stx, and two *Escherichia* phages, including phi191 and Lys12581Vzw. Furthermore, Lys12581Vzw, isolated from an outbreak STEC O145 strain, shared high sequence similarity (84% coverage and 98% identity) to *Shigella* phages Ss-VASD, suggesting that Stx-converting phage had potentially infected different bacterial species. All of the Stx-converting phages, including Lys19259Vzw, in clade 2 and clade 3 were from various STEC O157 strains isolated from different sources, such as river and clinical samples; the results likely indicated the diversity of Stx-converting phages from diverse STEC O157 strains.

**FIGURE 3:**
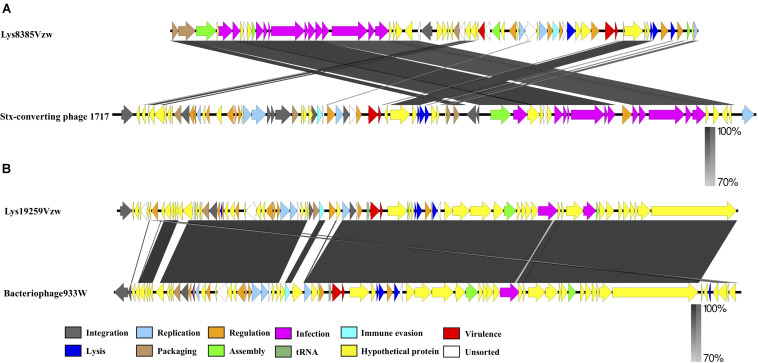
Whole-genome comparison of the Stx-converting phages Lys8385Vzw **(A)** and Lys19259Vzw **(B)** with the phylogenetically related Stx-converting phages 1717 and 933W, respectively, using BLASTn and visualization with EasyFig. Arrows represent annotated CDSs of each phage genome (top: phages Lys8385Vzw and Lys19259Vzw; bottom: phages 1717 and 933W), and biological functions of the CDSs are coded with different colors in the legend. Regions of sequence similarity, ranging from 70 to 100%, are connected by a gray-scale shaded area.

Comparative genomics of Stx1-converting phage Lys8385Vzw and Stx2-converting phage 1717 showed that the two phages had approximately an 11 kb difference in the length (Figure 3A), which primarily contained several different regulatory genes, including *Cro*, *CI*, *CII*, *CIII*, and *N*, related to the lytic-lysogenic switch in the phage 1717 genome; these findings likely differentiate the lifecycle of these two Stx-converting phages. Phage Lys8385Vzw and 1717 also contained the CDSs found in most siphophages, which were annotated with the predicted functions associated with phage packaging, assembly, and infection, such as terminase, major capsid proteins, tail proteins, tail measure proteins, and portal proteins. However, comparative genomics for phages Lys19259Vzw and 933W indicated that most CDSs coding for proteins with known functions in Lys19259Vzw shared a high identity to their counterparts in bacteriophage 933W (Figure 3B). However, low nucleotide sequence similarity (<70%) between these two phages was observed in several CDSs encoding the proteins, such as a few hypothetical proteins, integrase *intA*, and two phage anti-repressor protein *Ant*. Moreover, phage Stx2-converting phage Lys19259Vzw contained the *stx2* gene and the regulatory genes associated with the production of Stx in bacteria, which were 100% identical in nucleotide sequence to their respective counterparts in the phage 933W, suggesting that Lys19259Vzw might have similar capabilities as the well-characterized phage 933W in contributing to the development of bacterial pathogenicity (Del Cogliano et al., 2018).

### Genomic Differences Between the Stx Prophages Predicted and the Stx-Converting Phages Induced From the Same Bacterial Hosts

**FIGURE 4:**
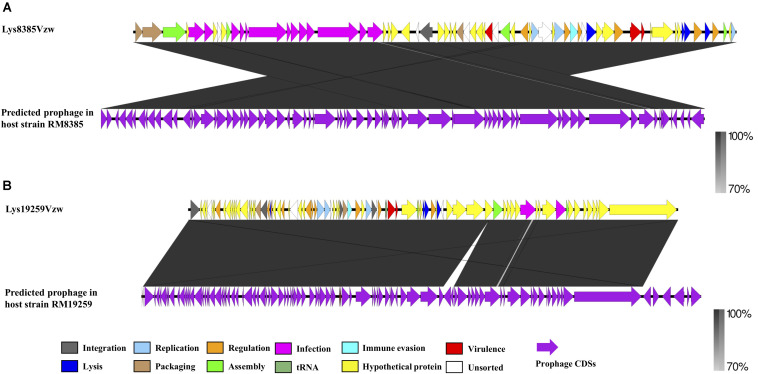
Pairwise comparison of the Stx-converting phages Lys8385Vzw **(A)** and Lys19259Vzw **(B)** with the corresponding Stx prophage sequences obtained from the bacterial genomes of *E. coli* RM8385 and *E. coli* RM19259, respectively, using the PHASTER web server. Arrows represent annotated CDSs, and the biological functions of the CDSs for the Stx-converting phages Lys8385Vzw and Lys19259Vzw (top) are coded with different colors in the legend. All CDSs are coded with purple for the Stx prophages (bottom). Regions of sequence similarity, ranging from 70 to 100%, are connected by a gray-scale shaded area.

To determine the change in nucleotide sequences that occurred during the prophage induction process, two Stx prophage sequences predicted from the genomes of *E. coli* O103:H11 (RM8385) and *E. coli* O157:H7 (RM19259), one prophage of each bacterial strain, were used to compare with Stx-converting phages Lys8385Vzw and Lys19259Vzw, respectively (Figure 4). According to the blastn results, the genomic structure of Stx-converting phage Lys8385Vzw was rearranged into two parts compared with the corresponding Stx prophage sequence, and the CDSs located in the first part encoded the proteins primarily related to infection, assembly, and packaging mechanisms; those in the second part were associated with virulence, regulation, cell lysis, and phage replication (Figure 4A). Additionally, the packaging mechanisms of Lys8385Vzw belonged to the cos phage with the 3′ cohesive ends, and the cos site (CGCCCCCCCT) was identified at the end of Lys8385Vzw genome (sequence position, base pairs 50,977– 50,953). In contrast, the blastn results showed that Stx-converting phage Lys19259Vzw shared 100% nucleotide sequence identity of the corresponding Stx prophage sequence (Figure 4B). Interestingly, the packaging mechanism of phage Lys19259Vzw was identified as Mu-like class via PhageTerm in that the phage DNA termini corresponded to fragments of the host DNA (Garneau et al., 2017). Furthermore, the predicted Stx2 prophage within RM19259 contained seven more CDSs than that in the Lys19259Vzw genome. Two CDSs coding for IS3 family transposase IS*Ec25* and IS3 family transposase IS*1203* were annotated in the middle of the prophage sequence (chromosome position of RM19259: base pairs 299,519–300,709) but were absent in phage Lys19259Vzw genome. The phenomenon likely indicated the occurrence of transposition during the prophage induction process. The other five CDSs located at the 3′ end of the prophage genome (chromosome position of RM19259: base pairs 338,419–343,278) were predicted to encode integrase, putative prophage DNA injection protein, DNA transfer protein, DUF2824 family protein, and phage_tail_NK domain-containing protein.

### Genome Comparison Between Stx-Converting Phages and Stx Prophages Predicted From STEC Strains From Different Sources

**FIGURE 5:**
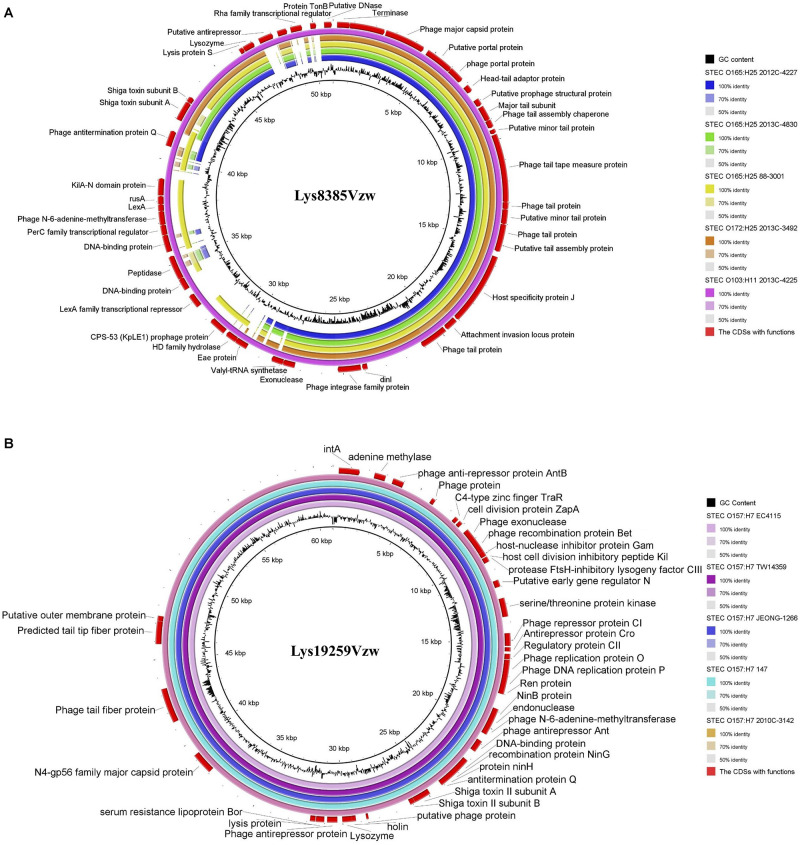
Genomic relationships between the Stx-converting phages Lys8385Vzw **(A)** and Lys19259Vzw **(B)** with five similar Stx prophages within STEC reference genomes. The color key coding for five circular maps of the bacterial sequence fragments with high sequence identity to the Stx-converting phage genomes is illustrated on the right, and the gradients of each color indicate the sequence identity, ranging from 50 to 100%, between Lys8385Vzw and its closely related phages **(A)** and between Lys19259Vzw and its closely related phages **(B)**. The CDSs with functions from the phages Lys8385Vzw and Lys19259Vzw are displayed in the outermost ring with red color. The BLASTn comparison of the phage sequences was generated using BLAST Ring Image Generator (BRIG) with a minimum nucleotide sequence identity of 50%.

Stx1-converting phage Lys8385Vzw and Stx2-converting phage Lys19259Vzw were compared with five selected reference STEC genomes to evaluate their correlations. Stx1-converting phage Lys8385Vzw shared more than 95% nucleotide identity over 71–100% coverage of the Stx prophage sequences within the five STEC strains, including *E. coli* O103:H11 (2013C-4225), *E. coli* O165:H25 (88-3001), *E. coli* O165:H25 (2012C-4227), *E. coli* O165:H25 (2013C-4830), and *E. coli* O172:H25 (2013C-3492). Lys8385Vzw shared the highest nucleotide sequence identity (99.86% identity and 100% coverage) to the Stx1 prophage from the STEC O103:H11 strain (2013C-4225) (Figure 5A). Additionally, the Stx2a prophage within *E. coli* O165:H25 strain (88-3001) shared 95.58% identity over 83% coverage of the nucleotide sequence of Stx-converting phage Lys8385Vzw but lacked some regulation-related genes, such as those coding for LexA family transcriptional repressor, and antitermination protein Q. Furthermore, Stx2-converting phage Lys19259Vzw shared both 99.99% nucleotide sequence identity and 99.99% coverage to the Stx prophage sequences obtained from five reference genomes of the STEC O157:H7 strains, including TW14359, EC4115, 2010C-3142, JEONG-1266, and 147 (Figure 5B). The results indicated the potential lysogenization of Stx-converting phages among various *E. coli* strains isolated from different samples, such as clinical specimens and environmental materials.

## Discussion

Various prophages are present in bacterial genomes containing some critical genes associated with bacterial pathogenesis and are considered as mobile genetic elements that play important roles in bacterial evolution (Castillo et al., 2018). Stx prophages embedded in bacterial genomes are highly related to the human pathogenicity of STEC strains, resulting in HUS with severe complications after infection (Krüger and Lucchesi, 2015). Because of the advancement of whole-genome sequencing technology, several studies have revealed the genomic evidence indicating that acquisition of additional Stx prophages is attributed to the enhanced pathogenicity of many outbreak-associated STEC strains (Quirós and Muniesa, 2017; Bai et al., 2019). However, the accuracy of the prophage screening and prediction, such as for Stx prophages, may vary based on the use of different computational tools. The results may also affect the downstream analyses regarding the phage-associated bacterial pathogenicity (Akhter et al., 2012; Arndt et al., 2017; Song et al., 2019). Previous studies have confirmed that Stx-converting phages, as free phages released from the STEC strains through Stx prophage induction, posed a risk of infecting susceptible *E. coli* strains and transferring *stx* genes to form new lysogens (Muniesa et al., 2004; Smith et al., 2012; Zhang et al., 2020). Therefore, the study of induced phages, such as Stx-converting phages, is important for understanding the phage-mediated bacterial pathogenicity and the emergence of new STEC pathogens. However, the information regarding the number of genomically characterized Stx-converting phages in the public database is scarce. In this study, two Stx-converting phages—Stx1-converting phage Lys8385Vzw and Stx2-converting phage Lys19259Vzw—induced from *E. coli* O103:H11 (RM8385) and *E. coli* O157:H7 (RM19259) strains, respectively, were subjected to whole-genome sequencing and genomic characterization to understand the role of these Stx-converting phages associated with different STEC strains.

The International Committee on Taxonomy of Viruses (ICTV) provides recommendations for classifying phages based on morphology and nucleotide sequences (Lefkowitz et al., 2018). In this study, the phylogenetic results aligned with the morphologies of the 15 selected Stx-converting phages. Additionally, the results of comparative genomics among these phages demonstrated the high homologies of the genes encoding phage assembly and infection-associated proteins, such as major capsid proteins, portal proteins, structural proteins, and tail proteins. Several studies have also confirmed that the genes encoding structural proteins, such as major capsid and tail fiber proteins, were the primary conservative genes in the phage genomes (Adriaenssens et al., 2017; Peng and Yuan, 2018). A multilocus sequence typing for the genome of 70 Stx-converting phages, induced from STEC isolates, found that approximately 70% short-tailed phages shared a highly conserved tail fiber gene (Smith et al., 2007b). Furthermore, tail fiber proteins were related to the recognition of bacterial receptor proteins on the bacterial membrane for successful phage infection (Yosef et al., 2017; Prevelige and Cortines, 2018; Yehl et al., 2019). Another study conducted by Smith et al. (2007a) observed that short-tailed Stx-converting phages, such as Stx2-converting phage vB_EcoP_24B (also known as Φ24B), could recognize the same bacterial outer membrane YaeT (currently called BamA) via the phage tail fiber proteins. The phylogenetic analysis of this study also showed that all the short-tailed Stx-converting phages contained tail fiber genes sharing a high nucleotide identity to that in phage Φ24B (Supplementary Figure S2A). Although siphophage Lys8385Vzw genome did not contain the similar tail fiber genes, the gene with the predicted function of host specificity J protein, involved in the phage attachment of the host receptor and the induction of phage DNA ejection, was identified in the Lys8385Vzw, sharing 88% nucleotide sequence identity over 100% coverage with the counterpart in phage 1717 (data not shown) (Roessner and Ihler, 1984). Thus, the results indicated that the long-tailed Stx-converting phages—belonging to the *Siphoviridae* family—had different functional tail proteins to recognize the host receptors. These findings suggest a strong correlation between phage conservative tail proteins and the phage adsorption capability, subsequently resulting in successful Stx-converting phage lysogenization.

The integrase gene plays an important part in the lysogenic cycle of lysogenic phage (Balding et al., 2005). Phage integrase is an enzyme associated with integrating a phage genome into the bacterial chromosomes at certain attachment sites (attP and attB) through a site-specific recombination process (Groth and Calos, 2004; Colavecchio et al., 2017). A previous study indicates that the genetic diversity of the integrase-coding genes among various Stx prophages was related to the different insertion sites of these prophages within the bacterial chromosome (Smith et al., 2007b). The results in this study showed that the predicted Stx1a prophage was integrated into the *dusA* site, encoding tRNA dihydroxyuridine synthase A, within the genome of RM8385 strain, whereas the predicted Stx2a prophage was integrated into the tRNA-*Arg* site in the bacterial genome of RM19259 strain. The results of the phylogenetic analysis showed that Stx1-converting phage Lys8385Vzw had a unique integrase gene as compared to other Stx-converting phages in this study (Supplementary Figure S2B). The same insertion site *dusA* was also found in novel Stx2k prophages embedded in three STEC strains isolated from patients and raw beef in China (Yang et al., 2020). Furthermore, a correlation between integrase sequences and phage insertion sites within the bacterial host chromosome was studied (Steyert et al., 2012; Yin et al., 2015). A previous study demonstrated that the phage insertions were governed by the phage integrases among Stx prophages in the locus of enterocyte effacement–negative STEC strains, because these Stx prophages carried the identical integrases utilizing the same insertion site (Steyert et al., 2012). Another study showed that distinct phage sequence types (PSTs)—PST1, PST2, and PST3—were correlated with different insertion sites (Yin et al., 2015). The authors found the phages in PST1 cluster all inserted in the *wrbA* site, encoding a TrpR binding protein, whereas those in both PST2 and PST3 clusters, containing the identical *int* sequences, were inserted in the tRNA-*argW* site. These findings are consistent with the present results that the Stx prophages similar to phages Lys8385Vzw and Lys19259Vzw could use the same insertion site (*dusA* and tRNA-*Arg*) in their corresponding bacterial strains of different origins. Specifically, Stx1 prophage of *E. coli* strain 2013C-4225 that shared identical integrase-coding sequence with Lys8385Vzw was also inserted in *dusA* gene, whereas the five Stx2 prophages that shared the identical integrase with phage Lys19259vzw were inserted into the tRNA-*Arg* site (data not shown). Importantly, contrary to the λ superinfection immunity model, previous studies have demonstrated that a number of STEC strains carried two or more Stx prophages within different insertion sites (Allison et al., 2003; Fogg et al., 2007; Serra-Moreno et al., 2007). Together, these findings reveal an important role of integrase in the lysogenic cycle, which is linked to the lysogenization of Stx-converting phages in the bacterial genomes, subsequently resulting in the evolution of STEC strains.

In the present study, Stx1-converting phage Lys8385Vzw and Stx2-converting phage Lys19259Vzw contained different numbers of tRNAs and regulatory protein-coding genes. Stx2-converting phage Lys19259Vzw contained three tRNA (two tRNAs-*Arg* and one tRNA-*Met*) in the phage genome. Interestingly, this study also found that tRNA-*Arg* was the most frequently found tRNA in the bacterial genome of RM19259 (data not shown). Previous studies indicated that tRNAs within the phage genome could compensate for differences in codon and/or amino acid usage between viruses and hosts to favor an efficient protein synthesis (Bailly-Bechet et al., 2007; Morgado and Vicente, 2019). Thus, tRNAs carried by lysogenic phages likely showed a codon usage bias close to the hosts, contributing to the protein synthesis of prophages and the trait acquisition of the hosts (Bailly-Bechet et al., 2007). Additionally, five regulatory protein-coding sequences, including phage repressor *CI*, antirepressor *Cro*, regulatory *CII*, antirepressor *CIII*, and early gene regulator *N*, were present in Stx2-converting phage Lys19259Vzw but absent in Stx1-converting phage Lys8385Vzw. The presence of these regulatory genes in both Stx1- and Stx2-converting phages was highly associated with regulation of Stx prophage induction process, triggering a lytic cycle of Stx prophages to lyse bacterial host and release Stx-converting phages in the environment (Smith et al., 2012; Steyert et al., 2012; Krüger and Lucchesi, 2015). These findings are consistent with our previous study that Lys19259Vzw had a higher induction efficiency than Lys8385Vzw regardless of the type of inducing factors likely due to a higher number of lytic-lysogenic switch genes in phage Lys19259Vzw than Stx1-converting phage Lys8385Vzw (Zhang et al., 2020).

The correlation between Stx prophage induction and Stx production, the primary virulence factors of STEC causing severe human disease, has been demonstrated in several studies (Zhang et al., 2000; Ethelberg et al., 2004; Chan and Ng, 2016). It has been showed that the expression of *stx* genes was regulated by the transcription of antiterminator Q and the late promoter p_*R*_, both located downstream of *stx* genes (Schmidt, 2001). In the current study, the phylogenetic results showed that the antitermination *Q* gene in Lys19259Vzw shared a high nucleotide identity with the counterfeit, also known as *Q*_933_, in phage 933W; the phage antitermination *Q* gene in phage Lys8385Vzw was different from other Stx-converting phages (Supplementary Figure S2C). A previous study reported that Certain STEC strains carrying the antiterminator *Q*_933_ gene within the Stx prophage sequence could produce higher levels of Stx than those harboring other types of antiterminator *Q* genes, such as *Q*_21_, within the Stx prophage sequence (LeJeune et al., 2004; Ahmad and Zurek, 2006). These findings infer that lysogenization of phage Lys19259Vzw, which carried the *Q*_933_ gene, may contribute to the high pathogenicity of the lysogen via producing a high level of Stx. However, the effects of the lysogenization of Stx-converting phage Lys8385Vzw on promoting Stx production from the new lysogen should be further investigated.

Because of the lysogenic capability of Stx-converting phages, the phages serve as mobile genetic elements contributing to the change of biological characteristics in bacteria; thus, understanding the functions of different genes present in the phage genome is important. In this study, among the genes predicted in the Stx-converting phages Lys8385Vzw and Lys19259Vzw, there were 11 types of functions identified as previously described (Song et al., 2019). These two Stx-converting phages contained genes coding for the functional proteins related to phage survival, such as lysis protein, terminase, and DNA-binding protein. These genes are commonly found in the genomes of most lytic and lysogenic phages and associated with phage DNA replication and packaging, lysis of bacterial cell membrane, and the release of phage progenies (Breschkin and Mosig, 1977; Bernhardt et al., 2000; Catalano, 2000; Xu, 2015; Garneau et al., 2017). Specifically, both Stx-converting phages in this study contain the genes encoding lysis protein, which is significantly expressed and accumulated in the bacterial cytoplasm at the end of the phage induction process (Moak and Molineux, 2004; Rodríguez-Rubio et al., 2013, 2016). The gene coding for phage N-6-adenine-methyltransferase was identified in both phages Lys8385Vzw and Lys19259Vzw, and the protein was highly involved in the process of phage infection to defend the bacterial restriction-modification system (Brooks and Roberts, 1982). Additionally, Stx1-converting phage Lys8385Vzw had the gene encoding the HD family hydrolase, which could bind to specific sites of the host bacterial cell membrane and subsequently disrupt the structure of the bacterial cell wall to facilitate the injection of phage DNA into the infected bacteria (Rodríguez-Rubio et al., 2016). Furthermore, both Stx-converting phages also carry some genes encoding proteins that are beneficial to their STEC hosts, including transcriptional repressors associated with the virulence gene expression, and the enhancement of STEC colonization in the gastrointestinal tract (Roberts et al., 1998; Nübling et al., 2014; Krüger et al., 2018). A previous study found that some lysogenic phages carried additional cargo genes associated with the difference of bacterial phenotype or bacterial fitness characteristics, such as colonization capability, rather than with the biological features of the phages (Brussow et al., 2004). These findings, along with those from the current study, indicate that Stx-converting phages contain two primary groups of genes: one group is associated with phage replication and the other group is related to the regulation of their bacterial hosts, particularly for bacterial pathogenicity.

Overall, this study provides the next-generation sequencing and genomic characterization of Stx1-converting and Stx2-converting phages, both induced from environmental STEC strains. The comparative genomics shows the genetic evidence regarding the potential dissemination of *stx* genes among the bacterial population via phage lysogenization. These findings provide additional information on the diversity of Stx-converting phages and contribute valuable insights into the role of Stx-converting phage in the evolution of bacterial pathogenicity through lysogenization in STEC strains. Additional studies are needed to confirm the predicted protein functions of the Stx-converting phages and the coevolution of Stx-converting phages with their STEC hosts.

## Data Availability Statement

The datasets presented in this study can be found in online repositories. The names of the repository/repositories and accession number(s) can be found below: https://www.ncbi.nlm.nih.gov/genbank/, MT225100; https://www.ncbi.nlm.nih.gov/genbank/, MT225101.

## Author Contributions

YZ performed phage induction and isolation, whole-genome sequencing, and genomic analysis and authored the manuscript. Y-TL performed genomic analyses and authored the manuscript. AS performed whole-genome sequencing. VW conceived and supervised the study, designed experiments, and reviewed drafts of the manuscript. All authors read and approved the final draft of the manuscript.

## Conflict of Interest

The authors declare that the research was conducted in the absence of any commercial or financial relationships that could be construed as a potential conflict of interest.

## Footnotes

1 https://blast.ncbi.nlm.nih.gov/Blast.cgi?PAGE=Nucleotides

2 https://blast.ncbi.nlm.nih.gov/Blast.cgi?PAGE=Proteins

3 https://blast.ncbi.nlm.nih.gov/Blast.cgi?PAGE=Nucleotides

## Supplementary Material

Supplementary Figure 1 Morphology of Stx-converting phages observed by transmission electron microscopy. **(A)** Phage Lys8385Vzw has a long and non-contractile tail. **(B)** Phage Lys19259Vzw has a short tail, composed of six short subterminal fibers.

Supplementary Figure 2 Maximum likelihood phylogenetic analysis of two Stx-converting phages, Lys8385Vzw and Lys19259Vzw (highlighted with blue font), and 13 reference Stx-converting phages from the NCBI database. **(A)** Tail fiber, **(B)** integrase, **(C)** antitermination *Q*. No predicted gene coding for tail fiber was found in the genome of phage Lys8385Vzw. The scale bar represents the percent divergence.
