# Supplementary figures and images for: Genomic Characterization of Two Shiga Toxin–Converting Bacteriophages Induced From Environmental Shiga Toxin–Producing Escherichia coli
Source: Front Microbiol. 2021 Feb 25;12:587696. doi: 10.3389/fmicb.2021.587696 (PMC7946995; doi:10.3389/fmicb.2021.587696)

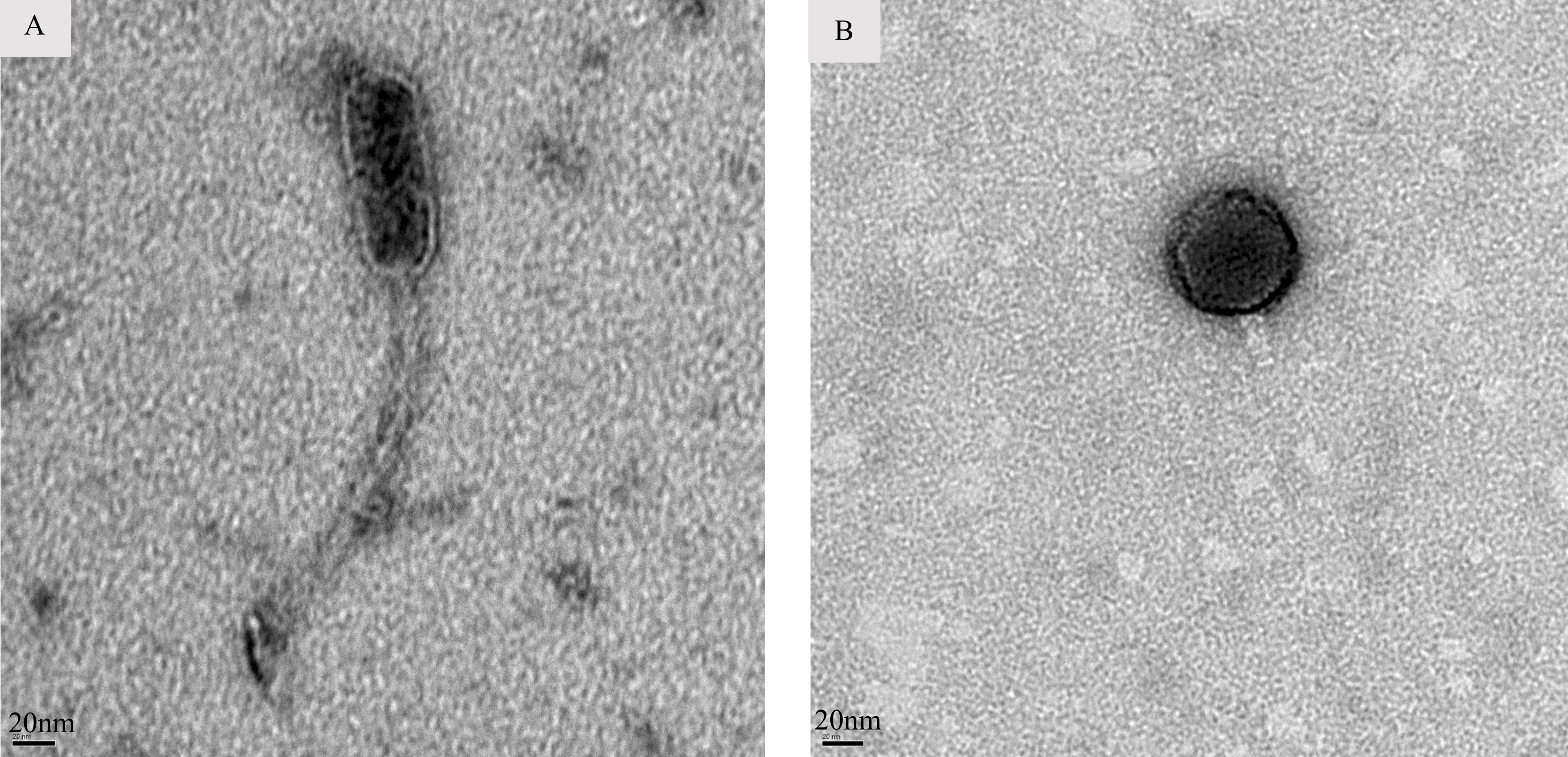

Supplement: Supplementary Figure 1 — Morphology of Stx-converting phages observed by transmission electron microscopy. (A) Phage Lys8385Vzw has a long and non-contractile tail. (B) Phage Lys19259Vzw has a short tail, composed of six short subterminal fibers. [file Image_1.JPEG]

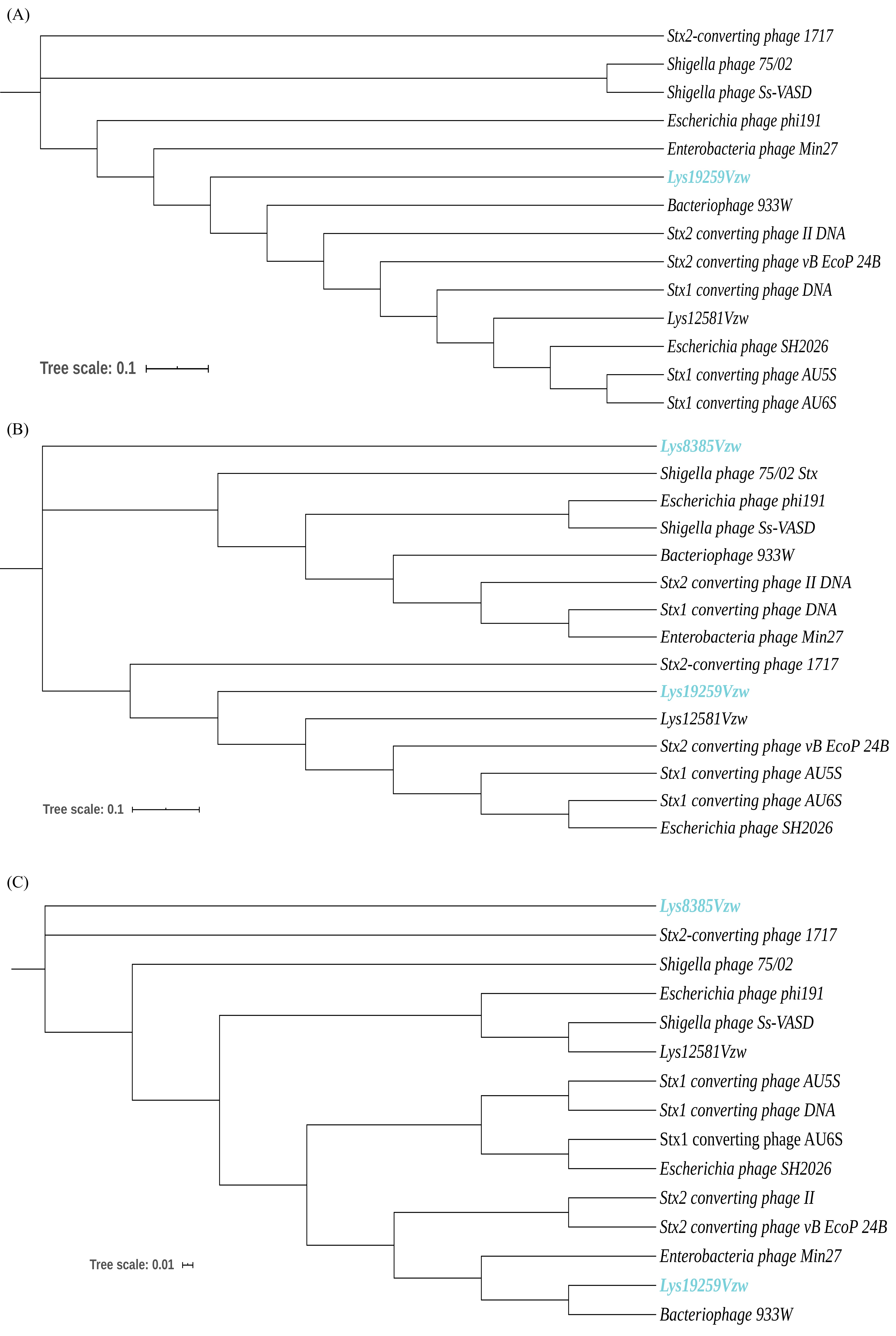

Supplement: Supplementary Figure 2 — Maximum likelihood phylogenetic analysis of two Stx-converting phages, Lys8385Vzw and Lys19259Vzw (highlighted with blue font), and 13 reference Stx-converting phages from the NCBI database. (A) Tail fiber, (B) integrase, (C) antitermination Q. No predicted gene coding for tail fiber was found in the genome of phage Lys8385Vzw. The scale bar represents the percent divergence. [file Image_2.JPEG]
