# Supplementary material for: Genomic Characterization of Two Shiga Toxin–Converting Bacteriophages Induced From Environmental Shiga Toxin–Producing Escherichia coli
Source: Front Microbiol. 2021 Feb 25;12:587696. doi: 10.3389/fmicb.2021.587696 (PMC7946995; doi:10.3389/fmicb.2021.587696)
Supplement: Supplementary file 3 [file Data_Sheet_1.PDF]

Supplementary Material

Table S1. The information of GenBank accession number, morphology, and the host strains for the Stx-converting phages obtained from the NCBI database.

**GenBank accession number**	**Phage ID**	***stx* gene**[α]	**Phage morphology**	**Bacterial host IDs**[β]	**Host Serotype**	**Reference**
AF125520.1	Bacteriophage 933W	*stx2a*	*Podoviridae*	*E. coli* EDL933	O157:H7	(Plunkett et al., 1999)
EU311208.1	*Enterobacteria* phage Min27	*stx2a*	*Podoviridae*	*E. coli* Min27	O157:H7	(Su et al., 2010)
KF971864.1	*Escherichia* phage phi191	*stx2a*	*Podoviridae*	*E. coli* ED 191	O111:H2	(Grande et al., 2014)
MG986485.1	*Escherichia* phage SH2026Stx1	*stx1a*	*Podoviridae*	*E. coli* SH2026	O157:H7	(Duan et al., 2018)
MN067333.1	*Escherichia* phage Lys12581Vzw	*stx2a*	*Podoviridae*	*E. coli* RM12581	O145:H28	(Zhang et al., 2019)
KF766125.2	*Shigella* phage 75/02 Stx	*stx1a*	*Podoviridae*	*Shigella sonnei* 75/02	N/A	(Tóth et al., 2016)
KR781488.1	*Shigella* phage Ss-VASD	*stx1a*	*Podoviridae*	*Shigella sonnei*[#]	N/A	(Carter et al., 2016)
KU977419.1	Stx1 converting phage AU5Stx1	*stx1a*	*Podoviridae*	*E. coli*[#]	O157:H7	\[^]
KU977420.1	Stx1 converting phage AU6Stx1	*stx1a*	*Podoviridae*	*E. coli*[#]	O157:H7	\
NC_004913.3	Stx1 converting phage DNA	*stx1a*	*Podoviridae*	*E. coli* Morioka V526	O157:H7	(Sato et al., 2003)
AP005154.1	Stx2 converting ph age II DNA	*stx2a*	*Podoviridae*	*E. coli* Morioka V526	O157:H7	(Sato et al., 2003)
HM208303.1	Stx2 converting phage vB_EcoP_24B	*stx2a*	*Podoviridae*	*E. coli*[#]	O157:H7	(Smith et al., 2012)

FJ188381.1	Stx2-converting phage 1717	*stx2c*	*Siphoviridae*	*E. coli*	O157:H7	\

[α]The *stx* gene subtype is identified in the phage genome. [β]The strain from which the phage was induced. [#]The strain ID is not available. N/A means the host serotype does not apply. ^ The references are not available in NCBI database.

Carter, C. C., Fierer, J., Chiu, W. W., Looney, D. J., Strain, M., and Mehta, S. R. (2016). A novel Shiga toxin 1a-converting bacteriophage of *Shigella sonnei* with close relationship to Shiga toxin 2-converting pages of *Escherichia coli*. Open Forum Infect. Dis. (Vol. 3, No. 2). doi:10.1093/ofid/ofw079.

Duan, M., Hunter, S. S., Minnich, S. A., Fagnan, M. W., New, D. D., Hovde, C. J., et al. (2018). Complete genome sequence of broad host- range Shiga toxin-converting bacteriophage SH2026Stx1, isolated from *Escherichia coli* O157:H7. Genome Announc. 6(25). doi:10.1128/genomeA.00490-18

Grande, L., Michelacci, V., Tozzoli, R., Ranieri, P., Maugliani, A., Caprioli, A., et al. (2014). Whole genome sequence comparison of vtx2-converting phages from Enteroaggregative Haemorrhagic *Escherichia coli* strains. BMC Genomics. 15(1), 574. doi:10.1186/1471-2164-15-574.

Plunkett, G., Rose, D. J., Durfee, T. J., and Blattner, F. R. (1999). Sequence of Shiga toxin 2 phage 933W from *Escherichia coli* O157:H7: Shiga toxin as a phage late-gene product J. Bacteriol. 181(6), 1767-1778. doi: 10.1128/JB.181.6.1767-1778.1999

Sato, T., Shimizu, T., Watarai, M., Kobayashi, M., Kano, S., Hamabata, T., et al. (2003). Genome analysis of a novel Shiga toxin 1 (Stx1)-converting phage which is closely related to Stx2-converting phages but not to other Stx1-converting phages. J. Bacteriol. 185, 3966–3971. doi:10.1128/JB.185.13.3966-3971.2003

Smith, D. L., Rooks, D. J., Fogg, P. C. M., Darby, A. C., Thomson, N. R., McCarthy, A. J., et al. (2012). Comparative genomics of Shiga toxin encoding bacteriophages. BMC Genomics. 13(1), 1-10. doi:10.1186/1471-2164-13-311.

Su, L. K., Lu, C. P., Wang, Y., Cao, D. M., Sun, J. H., and Yan, Y. X. (2010). Lysogenic infection of a Shiga toxin 2-converting bacteriophage changes host gene expression, enhances host acid resistance and motility. Mol. Biol. 44(1), 54-66. doi:10.1134/S0026893310010085.

Tóth, I., Sváb, D., Bálint, B., Brown-Jaque, M., and Maróti, G. (2016). Comparative analysis of the Shiga toxin converting bacteriophage first detected in *Shigella sonnei*. Infect. Genet. Evol. 37, 150-157. doi:10.1016/j.meegid.2015.11.022.

Zhang, Y., Liao, Y.-T., Salvador, A., Sun, X., and Wu, V. C. H. (2019). Complete Genome Sequence of a Shiga Toxin-Converting Bacteriophage, *Escherichia* Phage Lys12581Vzw, Induced from an Outbreak Shiga Toxin-Producing *Escherichia coli* Strain. Microbiol. Resour. Announc. 8, e00793-19. doi:10.1128/MRA.00793-19.

Table S2. The information of serotype, isolation source, and GenBank accession number for the Shiga toxin-producing *E. coli* (STEC) strains, including two strains used for prophage induction in this study and 10 reference STEC strains obtained from NCBI.

**Strain ID**	**Serotype**	***stx* gene**[α]	**Isolation Source**	**Accession Number**	**Reference**
RM8385[*]	O103:H11	***stx1a***	Cattle feces	NZ_CP028112.1	\[^]
2013C-4225	O103:H11	***stx1a***	Patient stool	NZ_CP027577.1	(Patel et al., 2018)
88-3001	O165:H25	***stx2a***, *stx2c*	Patient stool	CP027363.1	(Patel et al., 2018)
2012C-4227	O165:H25	***stx1a***, *stx2a*	Patient stool	NZ_CP013029.1	(Lindsey et al., 2015)
2013C-4830	O165:H25	***stx1a***, *stx2a*	Patient stool	NZ_CP027325.1	(Patel et al., 2018)
2013C-3492	O172:H25	***stx1a***, *stx2a*	Patient stool	CP027445.1	(Patel et al., 2018)
RM19259[*]	O157:H7	***stx2a***, *stx2c*	River	CP046527.1	(Zhang et al., 2020)
TW14359	O157:H7	***stx2a***, *stx2c*	Patient stool	NC_013008.1	(Kulasekara et al., 2009)
EC4115	O157:H7	***stx2a***, *stx2c*	Patient stool	NC_011353.1	(Eppinger et al., 2011)
2010C-3142	O157:H7	***stx2a***, *stx2c*	Patient stool	CP034801.1	\
JEONG-1266	O157:H7	***stx2a***, *stx2c*	Cattle feces	NZ_CP014314.1	(Teng et al., 2016)
147	O157:H7	***stx2a***	Cattle feces	CP028600.1	\

*The strains are used for Stx prophage induction in this study. ^ The references are not available in NCBI database. ᵅThe bold letters indicate the Stx prophage in bacteria sharing the high similarity with the genome of Stx-converting phage Lys8385Vzw or Lys19259Vzw.

Eppinger, M., Mammel, M. K., Leclerc, J. E., Ravel, J., and Cebula, T. A. (2011). Genomic anatomy of *Escherichia coli* O157:H7 outbreaks. Proc. Natl. Acad. Sci. U. S. A. 108(50), 20142-20147. doi:10.1073/pnas.1107176108.

Kulasekara, B. R., Jacobs, M., Zhou, Y., Wu, Z., Sims, E., Saenphimmachak, C., et al. (2009). Analysis of the genome of the *Escherichia coli* O157:H7 2006 spinach-associated outbreak isolate indicates candidate genes that may enhance virulence. Infect. Immun. 77(9), 3713-3721. doi:10.1128/IAI.00198-09.

Lindsey, R. L., Knipe, K., Rowe, L., Garcia-Toledo, L., Loparev, V., Juieng, P., et al. (2015). Complete genome sequences of two shiga toxin-producing *Escherichia coli* strains from serotypes O119:H4 and O165:H25. Genome Announc. 3(6). doi:10.1128/genomeA.01496-15.

Patel, P. N., Lindsey, R. L., Garcia-Toledo, L., Rowe, L. A., Batra, D., Whitley, S. W., et al. (2018). High-quality wholegenome sequences for 77 Shiga toxin producing *Escherichia coli* strains generated with PacBio sequencing. Genome Announc. 6(19). doi:10.1128/genomeA.00391-18.

Teng, L., Ginn, A., Jeon, S., Kang, M., and Jeong, K. C. C. (2016). Complete genome sequence of an *Escherichia coli* O157:H7 strain isolated from a super-shedder steer. Genome Announc. 4(2). doi:10.1128/genomeA.00258-16.

Zhang, Y., Liao, Y.-T., Salvador, A., Sun, X., and Wu, V. C. H. (2020). Investigating the Whole-Genome Sequence of a New Locus of Enterocyte Effacement-Positive Shiga Toxin-Producing *Escherichia coli* O157:H7 Strain Isolated from River Water. Resour. Announc. 9, e00112-20. doi: 10.1128/MRA.00112-20doi:10.1128/MRA.00112-20.

Table S3. The annotated coding DNA sequences (CDSs) with the predicted functions in Stx1-converting phage Lys8385Vzw genome.

**CDSs**	**CDS position**$^{\alpha}$ **(bp)**	**Length (bp)**	**Direction**	**Categories**$^{\beta}$
Terminase	185-793	609	forward	Packaging
Putative phage terminase, large subunit	790-2451	1662	forward	Packaging
Phage major capsid protein	2515-4452	1938	forward	Assembly
Putative portal protein	4664-6028	1365	forward	Infection

phage portal protein	6025-6792	738	forward	Infection
Head-tail adaptor protein	7095-7445	351	forward	Unsorted*
Putative prophage structural protein	7885-8229	345	forward	Assembly
Major tail subunit	8296-9012	717	forward	Infection
Phage tail assembly chaperone	9018-9392	375	forward	Infection
Putative minor tail protein	9488-9697	210	forward	Infection
Phage tail tape measure protein	9745-12987	3243	forward	Infection
Phage tail protein	12980-13321	342	forward	Infection
Putative minor tail protein	13321-14019	699	forward	Infection
Phage tail protein	14025-14768	744	forward	Infection
Putative tail assembly protein	14666-15343	678	forward	Assembly
Host specificity protein J	15589-19065	3477	forward	Infection
Attachment invasion locus protein	19132-19731	600	forward	Infection
Phage tail protein	19796-21109	1314	forward	Infection
dinI	23864-24112	249	reverse	Unsorted
Phage integrase family protein	24174-25271	1098	reverse	Integration
Exonuclease	27293-27865	573	reverse	Packaging
Valyl-tRNA synthetase	27865-28431	567	reverse	Unsorted
Eae protein	29711-30322	612	reverse	Unsorted
HD family hydrolase	30313-30849	537	reverse	Unsorted

CPS-53 (KpLE1) prophage protein	30977-31801	825	reverse	Assembly
LexA family transcriptional repressor	32687-33340	654	reverse	Regulation
DNA-binding protein	33661-34245	585	forward	Replication
Peptidase	34242-35387	1146	forward	Unsorted
DNA-binding protein	35605-36423	819	forward	Replication
PerC family transcriptional regulator	36426-36914	489	forward	Regulation
Phage N-6-adenine-methyltransferase	36914-37567	654	forward	Immune evasion
LexA	37564-37890	327	forward	Regulation
rusA	37887-38276	390	forward	Unsorted
KilA-N domain protein	38296-39105	810	forward	Lysis
Phage antitermination protein Q	40649-41401	753	forward	Regulation
Shiga toxin I a subunit A	41969-42916	948	forward	Virulence
Shiga toxin I a subunit B	42926-43195	270	forward	Virulence
Lysis protein S	46337-46522	216	forward	Lysis
Lysozyme	46557-47090	534	forward	Lysis
Putative antirepressor	47364-48059	696	forward	Regulation
Bacteriophage lysis protein	48297-48755	459	forward	Lysis
Rha family transcriptional regulator	48932-49483	552	forward	Regulation
Cytoplasmic membrane protein TonB	49874-50155	282	forward	Assembly

Putative DNase	50529-50894	366	forward	Replication

$^{\alpha}$The positions of CDSs located in the genome of phage Lys8385Vzw. $^{\beta}$The 11 categories of the predicted biological functions for the annotated CDSs were based on Song et al. (2019) with minor modifications. *The function of CDS is not clearly classified.

Song, W., Sun, H. X., Zhang, C., Cheng, L., Peng, Y., et al. (2019). Prophage Hunter: an integrative hunting tool for active prophages. Nucleic acids research, 47(W1), W74-W80. doi:10.1093/nar/gkz380.

Table S4. The annotated coding DNA sequences (CDSs) with the predicted functions in Stx2-converting phage Lys19259Vzw genome.

**CDSs**	**CDS position$^{\alpha}$ (bp)**	**Length (bp)**	**Direction**	**Categories$^{\beta}$**
intA	322-1491	1170	forward	Integration
adenine methylase	2321-2947	627	reverse	Unsorted*
phage anti-repressor protein AntB	3384-4007	624	reverse	Regulation
C4-type zinc finger TraR	7404-7625	222	reverse	Unsorted
cell division protein ZapA	7724-7939	216	reverse	Unsorted
Phage exonuclease	8359-9039	681	reverse	Packaging
phage recombination protein Bet	9036-9821	786	reverse	Integration
host-nuclease inhibitor protein Gam	9827-10123	297	reverse	Regulation
host cell division inhibitory peptide Kil	10178-10342	165	reverse	Lysis
protease FtsH-inhibitory lysogeny factor CIII	10311-10475	165	reverse	Regulation
Putative early gene regulator N	11596-11979	384	reverse	Regulation

serine/threonine protein kinase	12635-13681	1047	reverse	Unsorted
Phage repressor protein CI	14606-15313	708	reverse	Regulation
Antirepressor protein Cro	15392-15619	228	forward	Regulation
Regulatory protein CII	15758-16054	297	forward	Regulation
Phage replication protein O	16087-17025	939	forward	Replication
Phage DNA replication protein P	17022-17723	702	forward	Replication
Ren protein	17720-18010	291	forward	Unsorted
NinB protein	18881-19297	417	forward	Integration
endonuclease	19290-19892	603	forward	Packaging
phage N-6-adenine-methyltransferase	19889-20416	528	forward	Immune evasion
phage antirepressor Ant	20864-21538	675	forward	Regulation
DNA-binding protein	22176-22898	723	forward	Replication
recombination protein NinG	22898-23503	606	forward	Integration
protein ninH	23500-23694	195	forward	Unsorted
antitermination protein Q	23687-24121	435	forward	Regulation
tRNA-Met	24563-24638	76	forward	tRNA
tRNA-Arg	24646-24722	77	forward	tRNA
tRNA-Arg	24736-24812	77	forward	tRNA
Shiga toxin II a subunit A	24903-25862	960	forward	Virulence
Shiga toxin II a subunit B	25874-26143	270	forward	Virulence
Phage major capsid protein E	28564-28689	126	forward	Assembly

holin	29271-29486	216	forward	Lysis
Lysozyme	29491-30024	534	forward	Lysis
Phage antirepressor protein	30298-30687	570	forward	Regulation
lysis protein	31024-31488	465	forward	Lysis
serum resistance lipoprotein Bor	31520-31813	294	reverse	Unsorted
N4-gp56 family major capsid protein	38048-39262	1215	forward	Assembly
Phage tail fiber protein	41412-43349	1938	forward	Infection
Predicted tail tip fiber protein	45864-47132	1269	forward	Infection
Putative outer membrane protein	47147-47425	279	forward	Assembly

[α]The positions of CDSs located in the genome of phage Lys19259Vzw. [β]The 11 categories of the predicted biological functions for the annotated CDSs were based on Song et al. (2019) with minor modifications. [*]The function of CDS is not clearly classified.

Song, W., Sun, H. X., Zhang, C., Cheng, L., Peng, Y., et al. (2019). Prophage Hunter: an integrative hunting tool for active prophages. Nucleic acids research, 47(W1), W74-W80. doi:10.1093/nar/gkz380.

Figure S1. Morphology of Stx-converting phages observed by transmission electron microscopy. (A) Phage Lys8385Vzw has a long and non-contractile tail. (B) Phage Lys19259Vzw has a short tail, composed of 6 short subterminal fibers.

Figure S2. Maximum likelihood phylogenetic analysis of two Stx-converting phages, Lys8385Vzw and Lys19259Vzw (highlighted with blue font), and 13 reference Stx-converting phages from the NCBI database. (A) tail fiber, (B) integrase, (C) antitermination *Q*. No predicted gene coding for tail fiber was found in the genome of phage Lys8385Vzw. The scale bar represents the percent divergence.
